# Isoniazid-resistant TB and associated factors in Ethiopia

**DOI:** 10.5588/pha.25.0002

**Published:** 2025-06-04

**Authors:** S. Moga, T. Abebe, K. Bobosha, A. Alemu, G. Diriba, K.R.V. Harrington, R.H. Lyles, H.M. Blumberg, R.R. Kempker

**Affiliations:** ^1^ Addis Ababa University (AAU), Department of Microbiology, Immunology, and Parasitology, School of Medicine, College of Health Sciences, Addis Ababa, Ethiopia;; ^2^ Ethiopian Public Health Institute (EPHI), Infectious Diseases Research Directorate, Addis Ababa, Ethiopia;; ^*3*^ Armauer Hansen Research Institute (AHRI), Mycobacterial Diseases Research Directorate, Addis Ababa, Ethiopia;; ^4^ Emory University Rollins School of Public Health, Department of Epidemiology, Atlanta, GA, USA;; ^5^ Emory University Rollins School of Public Health, Department of Biostatistics and Bioinformatics, Atlanta, GA, USA;; ^6^ Emory University Rollins School of Public Health, Departments of Epidemiology and Global Health, Atlanta, GA, USA;; ^7^ Emory University School of Medicine, Department of Medicine, Division of Infectious Diseases, Atlanta, GA, USA.

**Keywords:** tuberculosis, *M. tuberculosis*, drug-resistant TB, MDR-TB, DST

## Abstract

**BACKGROUND:**

Isoniazid-resistant, rifampicin-susceptible *Mycobacterium tuberculosis* (Hr-TB) is the most common form of drug-resistant TB (DR-TB). We investigated the prevalence of and risk factors for Hr-TB in Ethiopia.

**METHODS:**

A cross-sectional study was conducted to determine the magnitude of Hr-TB, and to compare characteristics of persons with Hr-TB to those with multidrug-resistant TB (MDR-TB) and INH/RMP-susceptible TB identified during the National Drug Resistance Survey from 2017–2019.

**RESULTS:**

Among 1927 *M. tuberculosis* isolates recovered from persons with pulmonary TB, the prevalence of Hr-TB was 4.1% (95% CI 3.2-5.1), whereas the prevalence of MDR-TB was 1.9%. (95% CI 1.3–2.6). Unlike MDR-TB, the occurrence of Hr-TB did not differ significantly between new and previously treated TB cases (*P* = 0.67). The prevalence of Hr-TB cases was high in the Amhara (8.0%, 95% CI 4.8–12.5) region and Addis Ababa (7.1%, 95% CI 3.4–13.0). The proportion of Hr-TB increased with age (OR 1.02, 95% CI 1.01–1.04; *P* = 0.035). Compared to INH/RMP-susceptible TB, Hr-TB was more likely to harbor resistance to ethambutol, streptomycin and pyrazinamide (*P* < 0.0001).

**CONCLUSIONS:**

Hr-TB is the most prevalent type of DR-TB in Ethiopia and varies among regional states. Given the lack of identifiable clinical factors associated with Hr-TB, we recommend screening all bacteriologically confirmed TB cases for INH resistance at baseline.

Drug-resistant TB (DR-TB) remains a significant challenge to TB prevention and control.^1^ Isoniazid (INH) and rifampicin (RMP) are the two most important first-line drugs for the treatment of active TB disease. In 2023, INH-resistant TB (Hr-TB), which is defined as *Mycobacterium tuberculosis* (*Mtb*) strains resistant to INH but susceptible to RMP, occurred in an estimated one million persons with TB, accounting for 9.3% of estimated TB cases globally.^2^ Hr-TB poses a significant public health challenge as it diminishes the efficacy of first-line anti-TB treatments and increases the risk of progression to multidrug-resistant TB (MDR-TB), a form of TB that is resistant to RMP and INH.^3^ A systematic review showed that individuals with Hr-TB experience notably higher rates of treatment failure (11% vs 1%), TB relapse (10% vs 5%), and acquisition of further drug resistance during treatment (3.6% vs 0.6%) compared with persons with pan-susceptible TB.^3^ Moreover, a genomic study has revealed that the development of INH resistance is often the initial step towards acquiring additional resistance to other anti-TB drugs, including RMP and second-line drugs.^4^

Ethiopia is among 30 countries globally designated as high-burden TB and TB-HIV co-infection countries by the WHO.^2^ The COVID-19 pandemic has greatly impacted TB incidence and mortality, and reversed a decade-long trend of decline. In 2023, an incidence of 146 cases per 100,000 persons was reported, marking an increase compared to previous consecutive years.^2^ Although there are substantial data on MDR-TB, data describing Hr-TB remains more limited, particularly around identifying risk factors for Hr-TB in Ethiopia. The detection of INH resistance has historically received low priority, resulting in Hr-TB often being treated empirically with first-line TB drugs.^5^ This practice contributes to suboptimal treatment outcomes, prolonged infectiousness and emergence of MDR-TB.^3^ Given the rising global prevalence of Hr-TB and its potential to progress to MDR-TB ^3^, identifying risk factors associated with Hr-TB is crucial for developing effective TB control strategies. We therefore set out to assess the prevalence of Hr-TB and identify risk factors associated with Hr-TB by leveraging data from the National Drug Resistance Survey (DRS) conducted in Ethiopia from 2017–2019.

## METHODS

We conducted a population-based cross-sectional study using data from the Ethiopian National DRS carried out by the Ethiopian Public Health Institute (EPHI) from August 2017 to January 2019 at selected health facilities across all regional states. A probability-proportional to size (PPS) cluster sampling method was used to select health facilities where persons with TB seek care and were diagnosed using either the Xpert MTB/RIF assay or acid-fast bacilli (AFB) microscopy. A health facility reporting ≥30 smear-positive pulmonary TB cases annually formed its own cluster, while those with fewer cases were grouped into district-level clusters. A total of 1,237 clusters were derived from 4,503 health facilities available during the DRS. Using PPS, 67 clusters encompassing 217 health facilities were selected to recruit 30 cases per cluster. The sample size was determined to include 2,010 newly diagnosed TB cases, and all previously treated cases registered for treatment during the study period at selected health facilities. During the DRS, 2,560 bacteriologically confirmed new and previously treated pulmonary TB cases registered for treatment were consecutively enrolled in selected health centers after obtaining written informed consent or assent. Patients who had received treatment for more than one week at the time of enrolment and patients with extra-pulmonary TB were excluded. Socio-demographic, clinical, and behavioural data was collected at enrollment through interview and review of medical records. After enrollment, the Xpert MTB/RIF assay was performed for confirmation of TB and for determination of RMP resistance. A morning sputum sample was collected at health facilities and was transported to the nearest TB culture laboratories available at Regional Reference Laboratories (RRLs) and Referral Hospitals for culture isolation of *Mtb* using Löwenstein-Jensen medium and the BACTEC mycobacteria growth indicator tube (MGIT) 960 system (Becton Dickinson, Sparks, Maryland, USA).

Culture-positive *Mtb* isolates were tested with phenotypic drug susceptibility testing (DST) at the EPHI National TB Reference Laboratory (NTRL) located in Addis Ababa, Ethiopia. Phenotypic DST was performed using the BACTEC MGIT 960 system according to WHO-recommended critical concentrations of 0.1µg/ml for INH, 1.0µg/ml for RMP, 1.0µg/ml for streptomycin (SM), 5µg/ml for ethambutol (EMB), and 100µg/ml for pyrazinamide (PZA).^6^ Whole genome sequencing (WGS) was also performed on culture isolates from patients with MDR-TB, Hr-TB, and selected INH/RIF-susceptible *Mtb* isolates to confirm DST results. Resistance was interpreted based on the 2023 WHO catalogue of mutations associated with drug resistance.^7^ In cases where phenotypic DST was not available, a GenoType MTBDR*plus* line probe assay (LPA) was utilized to detect additional INH and RMP resistance in smear-positive sediments.

Participant characteristics were summarised descriptively, overall and separately for the groups presenting with Hr-TB, MDR-TB, and INH/RMP-susceptible TB. Distributions of risk factors among the Hr-TB were compared against those in the INH/RMP-susceptible and the MDR-TB groups, using chi-square tests for categorical variables and two sample t tests for continuous variables. In instances where categorical variables had small cell counts, the Fisher’s exact test was used in place of chi-squared test. Stepwise logistic regression with entry and retention significance levels of 0.20 was applied to obtain parsimonious multivariable models for Hr-TB and MDR-TB status, using the set of risk factors that were examined univariately to form the list of candidate predictors. All statistical analyses were performed using the SAS statistical software package (SAS Institute, Inc.). Cases with RMP-resistant but INH-susceptible TB including RMP monoresistant TB were excluded from risk factor analysis. INH/RMP-susceptible cases include cases susceptible to both INH and RMP.

This study was reviewed and approved by the Institutional Review Boards (IRBs) of the Ethiopian Public Health Institute (EPHI-IRB-258-2020) and Addis Ababa University (041/21/DMIP). Because the study involved secondary analysis of DRS data, a waiver of individual informed consent was obtained from the IRBs. Informed consent was collected from participants enrolled in the primary DRS.

## RESULTS

### Characteristics of study participants

There were 2,560 pulmonary TB cases enrolled in the DRS; 2,148 cases were diagnosed through AFB smear-positive results and 412 cases by positive Xpert MTB/RIF results. Among these TB cases, 1,927 (75.3%) had DST results for at least RMP and INH. The remaining 633 cases were excluded from analyses due to lack of DST data for INH. The flow chart outlining the enrolment process in this study is shown in the Figure. Among 1927 participants, more than half (57.2%) were male, with a mean age of 30.0 years (standard deviation 13.4, range 2–99). More than one-third of cases reported no formal education (37.9%) and lived in an urban area (37.2%). Thirty-one (1.6%) participants had a prior history of taking INH preventive therapy. Additionally, 183 (9.5%) participants had a history of previous TB treatment. The demographic and clinical characteristics of study participants are presented in Table 1. Of the 1,927 *Mtb* isolates analysed, 79 (4.1%) were classified as Hr-TB. Among 79 Hr-TB cases, 61 were identified through phenotypic DST, 16 were detected using LPA, and 2 were additionally confirmed by WGS. The highest proportions of Hr-TB cases were observed in the Amhara (8.1%) regions and Addis Ababa (7.5%) (Table 1). Additionally, 36 (1.9%) isolates were identified as MDR-TB and 5 (0.3%) isolates exhibited RMP-resistant but INH-susceptible TB. Higher prevalence of MDR-TB (n = 5, 3.9%) was recorded in Addis Ababa, while only one (0.5%) case of MDR-TB was reported from Amhara region (Table 1). Overall, 115 (5.9%) isolates demonstrated INH resistance, including those that were RMP-susceptible and RMP-resistant. In contrast, INH/RMP-susceptible TB accounted for 93.8% (1807) of isolates.

**FIGURE.**
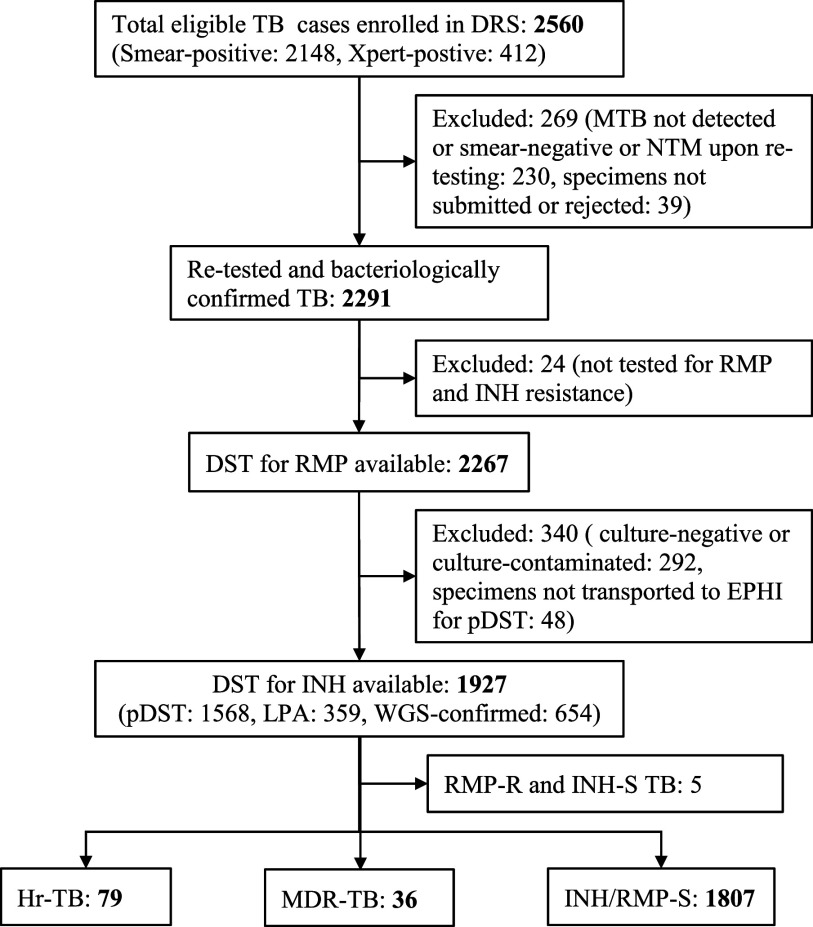
Flow chart of participant enrolment and isoniazid resistance pattern. DRS = drug resistance survey; NTM = nontuberculous mycobacteria; RMP = rifampicin; INH = isoniazid, DST = drug susceptibility testing; LPA = line probe assay: EPHI = Ethiopian Public Health Institute: RMP-R and INH-S TB= rifampicin resistant and isoniazid susceptible TB; Hr-TB = isoniazid-resistant, rifampicin-susceptible TB; MDR-TB = multidrug-resistant TB; INH/RMP-S TB = isoniazid and rifampicin susceptible TB

**TABLE 1.** Characteristics of participants, and comparison of isoniazid-resistant TB and isoniazid/rifampicin-susceptible TB.*

Characteristics		All cases (n=1927) n (%)	Hr-TB (N = 79) n (%)	INH/RMP-S TB (N = 1,807) n (%)	OR (95% CI) multivariable stepwise regression	*P*-value
Sex	Male	1,103 (57.2)	50 (63.3)	1,031 (57.1)		.272
	Female	824 (42.8)	29 (36.7)	776 (42.9)		
Age (Years)†	Mean (±SD)	30.0 ±13.4	33.1±15.6	29.9±13.3	1.02 (1.01–1.04)	0.01
Residence	Rural	1211 (62.8)	50 (63.3)	1,141 (63.1)		.979
	Urban	716 (37.2)	29 (36.7)	666 (36.9)		
Regional state‡	Oromia	666 (34.6)	25 (31.6)	633 (35.0)	2.32 (0.80, 6.81)	.004
	Amhara	237 (12.3)	19 (24.1)	217 (12.0)	5.54 (1.83, 16.75)	
	SNNPR	637 (33.1)	21 (26.6)	600 (33.2)	2.12 (0.72, 6.30)	
	Addis Ababa	141 (7.3)	10 (12.7)	124 (6.9)	4.05 (1.23, 13.27)	
	Others	246 (12.8)	4 (5.1)	233 (12.9)	Ref	
Education status	Primary	731 (37.9)	34 (43.0)	635 (35.1)	2.29 (1.27, 4.14)	0.02
	Secondary & higher	688 (35.7)	21 (26.6)	475 (26.3)	1.82 (0.93-3.56)	
	Illiterate	508 (26.4)	24 (30.4)	697 (38.6)	Ref	
Household family size (number)	≤ 5	1,318 (68.5)	53 (67.1)	1,239 (68.6)		.782
	>5	607 (31.5)	26 (32.9)	568 (31.4)		
TB treatment history	New	1,744 (90.5)	71 (89.9)	1,649 (91.3)		.671
	Previously treated	183 (9.5)	8 (10.1)	158 (8.7)		
HIV status	Reactive	142 (7.4)	6 (7.6)	130 (7.2)		.858
	Non-reactive	1,766 (91.7)	71 (89.9)	1,663 (92.0)		
	Unknown	19 (0.9)	2 (2.5)	14 (0.8)		
Current smoker	Yes	139 (7.2)	5 (6.3)	134 (7.4)		.718
	No	1,788 (92.8)	74 (93.7)	1,673 (92.6)		
Previous smoker	Yes	147 (7.6)	6 (7.6)	138 (7.6)		.989
	No	1,780 (92.4)	73 (92.4)	1,669 (92.4)		
History of incarceration	Yes	65 (3.4)	3 (3.8)	59 (3.3)		.743
	No	1,862 (96.6)	76 (96.2)	1,748 (96.7)		
Chewing khat	Yes	340 (17.6)	8 (10.1)	323 (17.9)		.076
	No	1,587 (82.4)	71 (89.9)	1,484 (82.1)		
Use of IPT	Yes	31 (1.6)	2 (2.5)	27 (1.5)		.392
	No	774 (40.2)	34 (43.0)	717 (39.7)		
	Unknown	1,122 (58.2)	43 (54.4)	1,063 (58.8)		
History of contact with TB	Yes	553 (28.7)	18 (22.8)	524 (29.0)		.232
	No	1,374 (71.3)	61 (77.2)	1,283 (71.0)		
History of contact with MDR-TB	Yes	22 (1.1)	1 (1.3)	19 (1.1)		.577
	No	1,905 (98.9)	78 (98.7)	1,788 (98.9)		

Hr-TB = isoniazid-resistant, rifampicin-susceptible TB; MDR-TB = multidrug-resistant TB; INH/RMP-S TB = isoniazid and rifampicin susceptible TB; IPT = isoniazid preventive therapy; SNNPR = Southern nations and nationalities, and peoples region

* All two-group comparisons of categorical predictors were made via the chi-squared test of general association, or via Fisher’s exact test when small cell counts are encountered.

† The age comparisons are made via the pooled two-sample t test.

‡ others = Ethiopian regional states including Tigray, Afar, Ethio-Somali, Benishangul-Gumuz, Gambella, Harari, Dire Dawa.

### Drug resistance profiles of Hr-TB and Isoniazid/rifampicin-susceptible TB

Phenotypic DST was performed for 1,568 of *Mtb* culture isolates. Among these, 61 were Hr-TB phenotypes, 1,474 were INH/RMP-susceptible TB, and the remaining 33 were either MDR-TB or RMP-resistant TB. A comparison between the DST patterns of Hr-TB and IHN/RMP-susceptible isolates for SM, EMB, and PZA are shown in Table 3. Among the 61 Hr-TB phenotypes, 30 exhibited additional resistance to ≥1 first-line anti-TB drug, whereas 31 demonstrated monoresistance to INH. Additional resistance was observed for SM (n = 27), for PZA (n = 5), and for EMB (n = 5). Two Hr-TB isolates were resistant to both PZA and EMB. In contrast, among INH/RMP-susceptible isolates, we observed resistance to SM (n = 30), PZA (n = 17), and EMB (n = 11). We found that Hr-TB cases were significantly associated with increased resistance to SM, EMB, and PZA compared to INH/RMP-susceptible strains (*P* < 0.0001).

**TABLE 3.** Phenotypic drug susceptibility profiles of patients with Hr-TB and isoniazid/rifampicin-susceptible TB.

Drugs (total number of pDST performed)	Phenotypic DST result	Hr-TB n (%)	INH/RMP-S TB n (%)	Estimated odds ratio (95% CI)	*P*-value
Streptomycin (n = 1,535)	Resistant	27 (44.3)	30 (2.0)	38.2 (20.5–71.1)	<.0001
	Susceptible	34 (55.7)	1,444 (98.0)		
Ethambutol (n = 1,535)	Resistant	5 (8.2)	11 (0.7)	11.9 (4.0–35.3)	<.0001
	Susceptible	56 (91.8)	1,463 (99.3)		
Pyrazinamide (n = 1,444)	Resistant	5 (8.3)	17 (1.2)	7.3 (2.6–20.5)	<.0001
	Susceptible	55 (91.7)	1,367 (98.8)		

Hr-TB = isoniazid-resistant, rifampicin-susceptible TB; INH/RMP-S TB = isoniazid and rifampicin-susceptible TB; DST: drug susceptibility testing; 95 % CI = 95% confidence interval

### Factors associated with Hr-TB and MDR-TB

Risk factors for Hr-TB in multivariable analysis included increasing age in years (aOR 1.02, 95% CI 1.01–1.04), primary level of education (aOR 2.29, 95% CI 1.27–4.14), and residence in Addis Ababa (aOR 4.05, 95% CI 1.23–13.27) and the Amhara region (aOR 5.54, 95% CI 1.83–16.75) (Table 1). The primary risk factor for MDR-TB was previous TB treatment (aOR 6.66, 95% CI 3.33–13.32), see Table 2, with marginal adjusted associations identified between MDR-TB and variables capturing confirmed prior MDR-TB contact and region of residence.

**TABLE 2.** Comparison of multidrug-resistant TB and isoniazid/rifampicin-susceptible TB.*

Characteristics		MDR-TB (N = 36) n (%)	INH/RMP-S TB (N = 1807) n (%)	OR (95% CI) multivariable stepwise regression	*P*-value
Sex	Male	20	1,031 (57.1)		.857
	Female	16	776 (42.9)		
Age (Years)†	Mean	30.7	29.9±13.2		.697
Residence	Rural	19 (52.8)	1,141 (63.1)		.202
	Urban	17 (47.2)	666 (36.9)		
Regional state‡	Oromia	6 (16.7)	633 (35.0)	0.29 (0.10- 0.84)	.011
	Amhara	1 (2.8)	217 (12.0)	0.13 (0.02- 1.02)	
	SNNPR	15 (41.7)	600 (33.2)	0.67 (0.28- 1.59)	
	Addis Ababa	5 (13.9)	124 (6.9)	1.15 (0.37- 3.63)	
	Others	9 (25.0)	233 (12.9)	Ref	
Education status	Illiterate	9 (25.0)	697 (38.6)		.204
	Primary	17 (47.2)	635 (35.1)		
	Secondary & higher	10 (27.8)	475 (26.3)		
Household family size (number)	≤ 5	23 (63.9)	1,239 (68.6)		.55
	>5	13 (36.1)	568 (31.4)		
TB treatment history	New	21 (58.3)	1,649 (91.3)	6.66 (3.33-13.32)	<0.001
	Previously treated	15 (41.7)	158 (8.7)		
HIV status	Reactive	5 (13.9)	130 (7.2)		.115
	Non-reactive	30 (83.3)	1,663 (92.0)		
	Unknown	1 (2.8)	14 (0.8)		
Current smoker	Yes	0 (0.0)	134 (7.4)		0.09
	No	36 (100)	1,673 (92.6)		
Previous smoker	Yes	3 (8.3)	138 (7.6)		.752
	No	33 (91.7)	1,669 (92.4)		
History of incarceration	Yes	2 (5.6)	59 (3.3)		.336
	No	34 (94.4)	1,748 (96.7)		
Chewing khat	Yes	9 (25.0)	323 (17.9)		.271
	No	27 (75.0)	1,484 (82.1)		
Use of IPT	Yes	2 (5.6)	27 (1.5)		.187
	No	19 (52.8)	717 (39.7)		
	Unknown	15 (41.7)	1,063 (58.8)		
History of contact with TB	Yes	10 (27.8)	524 (29.0)		.873
	No	26 (72.2)	1,283 (71.0)		
History of contact with MDR-TB	Yes	2 (5.6)	19 (1.1)	4.77 (0.94- 24.25)	0.06
	No	34 (94.4)	1,788 (98.9)		

Hr-TB = isoniazid-resistant, rifampicin-susceptible TB; MDR-TB = multidrug-resistant TB; INH/RMP-S TB = isoniazid and rifampicin susceptible TB; IPT = Isoniazid preventive therapy; SNNPR = Southern nations and nationalities, and peoples region

* All two-group comparisons of categorical predictors were made via the chi-squared test of general association, or via Fisher’s exact test when small cell counts are encountered.

† The age comparisons are made via the pooled two-sample t test.

‡ others = Ethiopian regional states including Tigray, Afar, Ethio-Somali, Benishangul-Gumuz, Gambella, Harari, Dire Dawa

## DISCUSSION

In this population-based study, the overall prevalence of Hr-TB was 4.1%. This prevalence is notably lower than the reported global estimate of Hr-TB at 9.3 %.^2^ However, the prevalence of Hr-TB represents a large proportion of the DR-TB cases in Ethiopia, particularly when compared to the relatively low rate of RMP-resistant TB and MDR-TB.^2^ Notably, our findings suggest a decline in the overall Hr-TB prevalence compared to 6.1% (95% CI 4.5–8.0) prevalence from the first DRS conducted in Ethiopia from 2003–2005.^8^ This reduction may highlight the impact of the TB control program in decentralization of rapid molecular diagnostics for the detection of DR-TB at primary health care level and high treatment success rate among DR-TB cases compared to global estimates. Ethiopia has seen a consistent decline in DR-TB prevalence, resulting in its removal from of the list of 30 high MDR-TB burden countries in 2021.^9^

Prior studies in Ethiopia have reported Hr-TB prevalence ranging from 8.9% to 9.3% among pulmonary TB cases.^10,11^ However, these studies were small in size and were conducted in general and tertiary hospitals that typically manage more complicated DR-TB cases, which may have led to an overestimation of Hr-TB prevalence. Previous work has described that studies with limited sample sizes based in specific institutions can inflate reported MDR-TB prevalence.^12^ Moreover, the pre-2020 diagnostic algorithm that recommended DST specifically for patients at high risk of MDR-TB could result in inflation of DR-TB in those studies. A strength of our findings includes the use of the population-based DRS and performing DST for bacteriologically confirmed TB cases, as this is likely a better representative sample to estimate the true prevalence of Hr-TB in Ethiopia. This underscores the need for comprehensive national surveys to accurately assess DR-TB burden in low and middle income countries including Ethiopia.

In our study, the prevalence of Hr-TB was 4.1% among newly diagnosed TB cases and 4.4% among previously treated TB cases. These estimates are lower than Hr-TB prevalence among new and previously treated TB patients reported in both the first DRS and global estimates, but they align with recent findings from Kenya and Eritrea.^8,13–15^ A history of previous anti-TB treatment is a well-established risk factor for developing MDR-TB in Ethiopia.^12^ Consistent with this study, the previous surveys indicate that patients with previous history of anti-TB treatment are at high risk for MDR-TB compared to new TB cases.^8^ However, it is noteworthy that Hr-TB was not associated with previous treatment history in our findings. This suggests the need for routine screening for INH resistance regardless of a patient’s previous treatment history.

We found an increase in the proportion of Hr-TB with increasing age. This could be due to increase in risk of TB disease with increasing age.^2^ A study showed that INH monoresistance tends to occur in persons aged 15–64 years.^16^ In contrast, another study highlighted a lower odds of INH monoresistant TB among older age groups.^17^ Although other studies inconsistently reported diabetes,^18^ non-diabetes,^19^ female sex,^20^ male sex, homelessness,^17^ use of preventive therapy, previous history of treatment ^21^ as independent risk factors associated Hr-TB, few studies also failed to demonstrate any significant predictors for Hr-TB.^22^ These works further reveal that factors associated with developing Hr-TB may vary by settings and more work is needed to gain a better understanding of these risks from different clinical and epidemiological perspectives. We also observed that Hr-TB more commonly occurred in Addis Ababa and the Amhara region, compared to other regional states in Ethiopia. Geographic variation in Hr-TB prevalence has been observed in other studies.^20,23^ This may imply local spread of Hr-TB and highlight the potential impact of setting-specific interventions and importance of real time molecular information to better identify and respond to local outbreaks.

We found association of Hr-TB with resistance to other first line drugs including EMB and PZA. This finding has raised important questions about which drug resistance appears first in the evolution of resistance. The most comprehensive genomic study of 5,310 Mtb isolates collected from five continents revealed that INH resistance emerge prior to RMP resistance and all other first-line drugs.^4^ Thus high proportion of resistance to first-line drugs observed among Hr-TB in our and other studies could partly explain the high rate of treatment failure and relapse rate among Hr-TB, compared to INH/RMP-susceptible TB.^3^ Moreover, a modelling study has indicated that INH resistance could even occur in an estimated 11% of latent TB infection (LTBI).^24^ This highlights that Hr-TB could emerge in TB patients without exposure to well-known predictors to DR-TB. This also implies that early detection of Hr-TB could prevent emergence of not only MDR-TB but also resistance to other first line TB drugs.

The Ethiopian national TB guidelines recommend rapid molecular testing for INH resistance among new and previously treated pulmonary TB cases that remain smear-positive at the second or later month of first line anti-TB drugs, to all previously treated cases at baseline, and to contacts of known Hr-TB cases.^5^ However, our data poses questions around whether targeted testing strategies or testing all TB patients for INH resistance is preferable. As we did not find any individual-level predictors compelling for targeted testing, our results suggest that a universal approach for INH-resistant screening should be considered. Consistently, the review of multi-country drug resistance data reported to WHO highlighted the need for INH DST for all bacteriologically confirmed TB cases at baseline prior to treatment.^23^ Moreover, rapid detection of RMP and INH resistance at moment of diagnosis has been shown to be cost effective and improve public health outcome.^25^ This is could enable the program to use levofloxacin in the treatment regimen and prevent worse treatment outcomes among Hr-TB cases.^26^

A limitation to this study is that certain cases were excluded because of lack of a culture growth or lack of DST for INH. However, our use of a national survey presented an opportunity to analyze risk factors and estimate the magnitude of INH that can be generalizable to overall TB/DR-TB patients.

## CONCLUSIONS

The prevalence of Hr-TB in Ethiopia is twice as high as MDR-TB prevalence, but notably lower than the global prevalence of Hr-TB. The prevalence of Hr-TB increased with advancing age and varied by regional states in Ethiopia with the highest rates in Addis Ababa and the Amhara region. We recommend baseline screening for Hr-TB among all bacteriologically confirmed TB cases given the impact on clinical care and increased resistance to other first line drugs. Expanding our current understanding of Hr-TB is important to guide policy makers and clinicians regarding the application of rapid molecular DST for screening of Hr-TB cases.

## Supplementary Material


