# Supplementary material for: Isoniazid-resistant TB and associated factors in Ethiopia
Source: Public Health Action. 2025 Jun 4;15(2):76–81. doi: 10.5588/pha.25.0002 (PMC12143246; doi:10.5588/pha.25.0002)
Supplement: Supplementary file 1 [file pha25-0002_supplementarydata1.pdf]

Supplementary Table 1. Comparison of cases with isoniazid DST that were included in the study and cases with missing isoniazid DST that were excluded from the analysis

**Characteristic**		**Included (n = 1,927) N (%)**	**Excluded (n = 364) N (%)**	***P*-value**
Sex	Male	1103 (57.2)	199 (54.7)	0.364
	Female	824 (42.8)	165 (45.3)	
Age (Years)†	Mean (±SD)	30.0±13.4	32.7±15.3	0.001
Residence	Rural	1211 (62.8)	232 (63.7%)	0.746
	Urban	716 (37.2)	132 (36.3)	
Regional state‡	Oromia	666 (34.6)	174 (47.8)	0.000
	Amhara	237 (12.3)	38 (10.4)	
	SNNPR	637 (33.1)	80 (22.0)	
	Addis Ababa	141 (7.3)	5 (1.4)	
	Others*	246 (12.8)	67 (18.4)	
Education status	Illiterate	731 (37.9)	185 (50.8)	0.000
	Primary	688 (35.7)	119 (32.7)	
	Secondary & higher	508 (26.4)	60 (16.5)	
Household family size (number)	≤ 5	1319 (68.4)	266 (73.1)	0.079
	>5	608 (31.6)	98 (26.9)	
TB treatment history	New	1744 (90.5)	311 (85.4)	0.04
	Previously treated	183 (9.5)	53 (14.6)	
HIV status	Reactive	142 (7.4)	36 (9.9)	0.247
	Non-reactive	1768 (91.7)	325 (89.3)	
	Unknown	17 (0.9)	3 (0.8)	
Current smoker	Yes	139(7.2)	27(7.4)	0.890
	No	1788 (92.8)	337 (92.6)	
Previous smoker	Yes	147 (7.6)	32 (8.8)	0.448

	No	1780 (92.4)	332 (91.2)	
History of incarceration	Yes	65 (3.4)	7 (1.9)	0.146
	No	1862 (96.6)	357 (98.1)	
Chewing khat	Yes	340 (17.6)	95 (26.1)	0.000
	No	1587 (82.4)	269 (73.9)	
Use of IPT	Yes	31 (1.6)	12 (3.3)	0.001
	No	774 (40.2)	114 (31.3)	
	Unknown	1122 (58.2)	238 (65.4)	
History of contact with TB	Yes	553 (28.7)	116 (31.9)	0.222
	No	1374 (71.3)	248 (68.1)	
History of contact with MDR-TB	Yes	22 (1.1)	9 (2.5)	0.044
	No	1905 (98.9)	355 (97.5)	

DST= drug susceptibility testing; SNNPR = Southern nations and nationalities, and peoples region; IPT = Isoniazid preventive therapy: MDR-TB = multidrug-resistant TB
